# Antibiotic Resistance in Nosocomial Bacteria Isolated from Infected Wounds of Hospitalized Patients in Czech Republic

**DOI:** 10.3390/antibiotics9060342

**Published:** 2020-06-18

**Authors:** Milan Kolar, Pavel Cermak, Lenka Hobzova, Katerina Bogdanova, Katerina Neradova, Patrik Mlynarcik, Pavel Bostik

**Affiliations:** 1 Department of Microbiology, Faculty of Medicine and Dentistry, Palacky University, and University Hospital, 779 00 Olomouc, Czech Republic; milan.kolar@fnol.cz (M.K.); katerina.bogdanova@fnol.cz (K.B.); patrik.mlynarcik@upol.cz (P.M.); 2 Department of Microbiology, Thomayer Hospital, 140 59 Prague, Czech Republic; pavel.cermak@ftn.cz; 3 Institute of Clinical Microbiology, Charles University Medical School and University Hospital, 500 05 Hradec Kralove, Czech Republic; lenka.hobzova@fnhk.cz (L.H.); katerina.neradova@fnhk.cz (K.N.); 4 Faculty of Military Health Sciences, University of Defence, 500 01 Hradec Kralove, Czech Republic

**Keywords:** nosocomial, wound infection, resistance

## Abstract

Hospitalized patients with wounds face an increased risk of infection with multi-drug-resistant nosocomial bacteria. In this study, samples from almost 10,000 patients from big hospitals in Czech Republic with infected wounds were analyzed for the presence of bacterial pathogens. In 7693 patients (78.8%), bacterial etiological agents were identified. Members of the *Enterobacterales* (37.1%) and *Staphyloccus aureus* (21.1%) were the most prevalent pathogens. *Staphyloccus aureus* showed methicillin resistance in 8.6%. Almost half of the *Klebsiella pneumoniae* isolates were ESBL-positive and 25.6% of the *Enterobacter* spp. isolates were AmpC-positive. The third most prevalent *Pseudomonas aeruginosa* showed resistance to 19–32% of the antipseudomonal antibiotics tested. Based on the results, amoxicillin/clavulanic acid, ampicillin/sulbactam or piperacillin/tazobactam combined with gentamicin can be recommended for antibiotic treatment of infected wounds. Once the etiological agent is identified, the therapy should be adjusted according to the species and its resistance.

## 1. Introduction

Bacterial skin and soft tissues infections (SSTIs) include, in order of severity, various manifestations, such as impetigo, folliculitis, furuncle, carbuncle, erysipelas, cellulitis, fasciitis and myonecrosis. In addition, other similar infections, such as infected decubitus ulcers and leg ulcers, as well as bacterial infections of surgical wounds, which are often caused by *Staphylococcus aureus*, *Enterococcus* spp., members of the *Enterobacterales* order and Gram-negative non-fermenting bacteria, are usually recognized as a part of SSTIs [1]. At the same time, one of the most pressing issues in medical care worldwide is the increasing resistance of various bacterial strains to the antibiotic treatment [2]. This applies to the infected decubitus and leg ulcers, and bacterial infections of surgical wounds, where often the causative agents are multi-drug-resistant (MDR) bacteria. This can lead to a failure of the antibiotic therapy with all the negative effects on the overall clinical status. It means a higher mortality and shorter survival of the patients with infections caused by the MDR bacteria due to the development of complications, such as systemic infections, sepsis and septic shock [3,4]. Tumbarello et al. reported that the mortality of patients with systemic infections caused by Extended Spectrum Beta-Lactamase (ESBL)-positive enterobacteria reached 60% in cases with an inadequate antibiotic therapy, while in those where the appropriate antibiotic was used it decreased to 19% [5]. Trecarichi et al. showed that the mortality in patients with hematologic malignancies associated with bloodstream infections caused by MDR *Pseudomonas aeruginosa* strains was as high as 40%, while the susceptible strains of the same bacteria led to the death of 9% of the patients [6]. Another study has shown that there was a statistically significant difference between the adequacy of the antibiotic treatment and the mortality of patients suffering from ventilator-associated pneumonia. Thus, if the causative bacteria were resistant to the initial antibiotic therapy, the mortality reached 46%, while in case of susceptible bacterial strains it was 27% [7].

The therapy of infected decubitus and leg ulcers and bacterial infections of surgical wounds consists of the local application of antibiotics, but a systemic application is often warranted as well. That, however, bears a potential risk of the selection of the MDR bacterial species, which, in turn, leads to a lower effectivity of the antibiotic treatment with an increased morbidity and mortality in these infections [5,6,7]. One of the promising ways to find novel therapeutic approaches and address the problem of increased bacterial resistance, is to search for or develop novel antibacterial compounds. Among these, extracts from hops *Humulus lupulus* L., which are effective against multi-resistant Gram-positive bacteria, including the methicillin-resistant *S. aureus* (MRSA) and vancomycin-resistant enterococci (VRE), show promise [8,9].

In this study, samples from the infected decubitus ulcers, leg ulcers and surgical wounds of almost 10,000 patients were subjected to the microbiological analysis and identification of the level of bacterial resistance. This is the first study with such a large cohort of patients in this region. The results are used to define the therapeutic approaches to the antibiotic treatment, including the potential of application of hops extracts in those cases caused by the Gram-positive MDR bacteria.

## 2. Results

Samples from a total of 9762 patients with diagnoses of infected decubitus ulcers, leg ulcers and bacterial infections of surgical wounds were included in this study. In samples from 7693 patients (78.8%), bacterial etiologic agents were identified. Bacterial isolation was negative or non-significant in 21.2% of samples.

The isolated and identified bacterial species are shown in Table 1. The results show that there were 10,977 pathogens identified in total and the most prevalent etiological agent was *S. aureus* (21.1%). Gram-positive facultative anaerobic cocci (staphylococci, streptococci and enterococci) represent 44.6% of all bacterial etiological agents. Other important bacterial pathogens include members of the *Enterobacterales* (37.1%) and *P. aeruginosa* (12.7%).

When the susceptibility/resistance of the individual bacterial species to the currently used antibiotics was analyzed, the results showed the following (Table 2). Very alarming is the finding that almost half of the *K. pneumoniae* isolates (48.3%) were ESBL-positive and 25.6% of the *Enterobacter* spp. isolates were AmpC-positive. *P. aeruginosa* isolates (the third most prevalent bacteria) also showed various resistance phenotypes at dangerously high levels. Thus, more than 27% of those were resistant to ciprofloxacin and meropenem and 24.2% to piperacillin/tazobactam. The most frequently isolated *S. aureus* (21.1% of all samples) showed the MRSA phenotype in 199 samples (8.6%). Relatively high numbers of streptococci with the MLS_B_ phenotype (resistance to macrolides, lincosamide and streptogramin B) are also worrisome. VRE were detected in 3.4% of all enterococci-positive cases, where 82.6% of those are represented by *E. faecium* with phenotype VanA and 17.4% by *E. faecalis* phenotype VanB. Relatively encouraging is the result that only 0.8% of the enterobacteria were resistant to carbapenems.

## 3. Discussion

The multicentric study presented herein analyzed the most prevalent bacterial strains found as causative agents in a cohort of almost 10000 patients from the Czech Republic suffering from infected decubitus ulcers, leg ulcers and bacterial infections of surgical wounds. Out of the total number of patients examined, 21% had clinical suspicion of infection with negative or insignificant bacterial culture results, but requiring antibiotic treatment with respect to the patient′s clinical condition. The reason for the negative or insignificant result could be the antibiotic treatment. A study with such a large cohort of patients has not been conducted in the Czech Republic to date and, moreover, it represents a pilot study from this European region. The results confirm that the most common bacterial strains in these infections are members of the *Enterobacterales* (37%), *S. aureus* (21%), *P. aeruginosa* (13%), *Enterococcus* spp. (12%) and *Streptococcus* spp. (11%). Our results are consistent with the study of Giacometti et al., who defined *S. aureus* (28%), *P. aeruginosa* (25%), *E. coli* (8%), *Staphylococcus epidermidis* (7%) and *E. faecalis* (6%) as the most frequent pathogens of wound infections in surgical patients [10]. Hedaoo et al. evaluated isolates from swabs/pus of surgical site infections and found that the most frequent ones were *Klebsiella* spp. (24%), *S. aureus* (20%), *E. coli* (15%) and *P. aeruginosa* (13%) [11].

The analysis of antibiotic resistance in the isolated bacterial pathogens confirms an alarming trend of increasing numbers of the MDR strains, namely of the enterobacteria producing the broad-spectrum beta-lactamases ESBL and AmpC, even in this large Czech cohort. Another upcoming serious problem is indicated by the finding of *P. aeruginosa* displaying resistance to selected antipseudomonal antibiotics within the range of 19–32%. In this cohort, MRSA and VRE also represented important pathogens, albeit with lower frequencies (9% and 3%, respectively). The high resistance of bacterial pathogens in infected surgical wounds is also confirmed by Hedaoo et al., who reports a 50% prevalence of MRSA. Additionally, more than 60% *E. coli* and *P. aeruginosa* showed resistance to gentamicin and more than 50% resistance to 3rd generation cephalosporins and ciprofloxacin [11]. The lower prevalence of MRSA in this study compared to other studies cannot be easily explained by the fact that hospitals differ in facilities and medical care approaches to their patients. One plausible explanation may be a differential utilization of beta-lactam antibiotics for the initial treatment.

The resistant bacteria in hospital settings are most commonly disseminated by the contaminated hands of healthcare workers and contaminated instruments or items of daily use [12]. Critically ill patients represent the most vulnerable group, in which the resistant bacteria cause serious and poorly treatable infections. This is a multi-factorial problem, which has to be solved using a complex approach. The basis of such an approach is formed by the rational application of antibiotics, as well as the implementation and adherence to proper epidemiological control measures with an active infection control approach aiming to identify resistant bacterial strains and limit their spread [13,14].

The results of the study presented herein can be used as a basis for antibiotic stewardship in infected decubitus ulcers, leg ulcers and surgical wounds. Antibiotic stewardship (AS) may be freely defined as a set of measures leading to rational antibiotic therapy based on adequate selection of antibacterial agents, relevant duration of their administration and a suitable route of administration. At the present time, it is essential to implement AS at all necessary levels. The AS system is rather comprehensive, containing a range of individual programs and activities, including (1) an adequate identification of bacterial pathogens in particular infections; (2) assessing the prevalence of pathogenic bacteria and their resistance to antibiotics; and (3) developing of local guidelines for the initial antibiotic therapy. Based on the obtained data, penicillin-type antibiotics with beta-lactamase inhibitors (amoxicillin/clavulanic acid, ampicillin/sulbactam or piperacillin/tazobactam) combined with gentamicin can be recommended for initial antibiotic therapy. However, the results of this study underscore even more the need to perform a proper bacterial analysis from the infected lesions as an integral part of the therapeutic approach. Once the etiological agent is identified, the therapy should be adjusted according to the species and, potentially, its resistance. This will lower the mortality and morbidity, speed-up the recovery and, maybe most importantly, prevent further selection of MDR bacterial species circulating in hospitals [5,6,7].

In this regard, the use of alternative/novel compounds with antimicrobial properties may present a suitable future direction for topical treatments. Our previous data suggest that extracts from hops, which showed strong antimicrobial properties against Gram-positive bacteria, including MRSA and VRE, may represent attractive alternatives for such treatments [8,9]. Due to the data above, showing that staphylococci, enterococci and streptococci represent 45% of the bacterial pathogens isolated from bacterial nosocomial infections of decubitus ulcers, leg ulcers and surgical wounds, together with the occurrence of MDR isolates among them, this may represent a viable option for future treatments. Another promising therapeutic option, particularly for infections caused by *Staphylococcus aureus*, including MRSA, is the use of natural antimicrobial peptides [15].

## 4. Materials and Methods

### 4.1. Patient Cohort

The study was performed in three large teaching hospitals in Czech Republic: the Faculty Hospital Olomouc (1212 beds), Faculty Hospital Hradec Kralove (1375 beds) and Thomayer Hospital in Prague (1063 beds), in patients hospitalized between January 1, 2016, and December 31, 2018. These three hospitals were intentionally selected so that their sizes (numbers of beds) and the extent of care were comparable. Data were obtained retrospectively from hospital information systems.

The bacteria were isolated from the swabs of the infected decubitus ulcers, leg ulcers and infected surgical wounds from a total of 9762 patients using standard microbiological methods, i.e., aerobic cultivation on appropriate agars (Blood agar, MacConkey agar, Trios, Czech Republic). The swabs were collected as part of the standard clinical care.

Case inclusion criterion was the development of infection during hospitalization, at least 48 h after the admission for hospitalization. No case exclusion criterion was applied. Only the first isolate of a bacterial strain from each patient within a given year was included in this study. The clinical significance of the isolated bacteria was determined based on the clinical and biochemical markers of the bacterial infection, the quantity of the bacterial pathogens and, eventually, the repeated detection of identical bacterial agents. Bacteria from the skin surface, in particular coagulase-negative staphylococci and corynebacteria, in small quantities were considered clinically non-significant.

### 4.2. Microbiological Analysis

The identification of bacteria was performed by MALDI-TOF MS (Biotyper Microflex, Bruker Daltonics, Bremen, Germany). The susceptibility/resistance to antibiotics was tested by the broth microdilution method according to EUCAST [16]. The following bacterial strains were used as references for the quality control: *Escherichia coli* ATCC 25922, *P. aeruginosa* ATCC 27853, *S. aureus* ATCC 29213 and *Enterococcus faecalis* ATCC 29212. The production of broad-spectrum beta-lactamases, such as ESBL and AmpC, was detected by phenotypic tests [17]. Positive results were verified by PCR detection of genes for the respective type of beta-lactamase. All strains of *S. aureus* were tested for the resistance to methicillin using selective diagnostic chromogenic media (Colorex/TM/MRSA, TRIOS, Prague, Czech Republic) and immunochromatographic assays for the detection of PBP2a (PBP2a SA Culture Colony Test, Alere^TM^, Abbott, Prague, Czech Republic). Positive results were confirmed by the detection of the *mecA* gene. The resistance to vancomycin in VRE was also confirmed by the detection of the *vanA* and *vanB* genes.

### 4.3. Molecular Biologic Analysis

The confirmation of the most prevalent mechanisms of the bacterial resistance to antibiotics was performed by the molecular analysis of the resistance genes. Thus, the PCR was performed using the reaction mixture containing 1 µL DNA (100 ng) in 24 µL complete reaction buffer with MgCl_2_ (containing 100 mmol Tris-HCl (pH 8.8), 500 mmol KCl, 1% Triton X-100, 15 mmol MgCl_2_) (Top-Bio, Czech Republic; 2.5 µL), dNTP (10 mmol, 0.5 µL), 50 µmol of each primer (0.2 µL) and Taq DNA polymerase (Top-Bio, Czech Republic; 0.2 µL). The PCR conditions were as follows: initial denaturation at 94 °C for 3 min, 30 cycles of 94 °C for 30 sec, at different annealing temperatures for 30 sec and 72 °C for 1 min, followed by a final elongation step at 72 °C for 10 min. The primer sequences and calculated lengths of the corresponding amplicons are listed in Table 3 [18,19,20,21,22,23].

Four reference strains were used as the positive controls: CTX-M, TEM-1 and OXA-1-positive *E. coli* (NCTC 13400), SHV-positive *Klebsiella pneumoniae* (NCTC 13368), VanA-positive *E. faecalis* (NCTC 12201) and MRSA (NCTC 13142).

## Figures and Tables

**Table 1:** Bacterial species isolated from infected decubitus ulcers, leg ulcers and surgical wounds.

Species	Number of Samples	Percent of Total
*Staphylococcus aureus*	2314	21.1
*Escherichia coli*	1623	14.8
*Pseudomonas aeruginosa*	1393	12.7
*Enterococcus faecalis*	1020	9.3
*Klebsiella pneumoniae*	899	8.2
*Streptococcus pyogenes*	724	6.6
*Proteus mirabilis*	680	6.2
*Enterobacter* spp.	636	5.8
*Enterococcus faecium*	329	3.0
*Streptococcus agalactiae*	285	2.6
*Morganella morganii*	230	2.1
*Streptococcus anginosus*	219	2.0
Other	625	5.7

**Table 2:** Bacterial species isolated from infected decubitus ulcers, leg ulcers and surgical wounds.

Resistance Phenotype	Number of Isolates	Percent of Total
Methicillin-resistant *Staphylococcus aureus*	199	8.6
Vancomycin-resistant enterococci	46	3.4
Streptococci of the MLS_B_ phenotype	183	14.9
ESBL-producing *Escherichia coli*	258	15.9
AmpC-producing *Enterobacter* spp.	163	25.6
ESBL-producing *Klebsiella pneumoniae*	434	48.3
Enterobacteria resistant to carbapenems	32	0.8
*Pseudomonas aeruginosa* resistant to meropenem	382	27.4
*Pseudomonas aeruginosa* resistant to ceftazidime	263	18.9
*Pseudomonas aeruginosa* resistant to ciprofloxacin	442	31.7
*Pseudomonas aeruginosa* resistant to gentamicin	274	19.7
*Pseudomonas aeruginosa* resistant to piperacillin/tazobactam	337	24.2

**Table 3:** Sequences of primers used for PCR for detection of resistance genes.

Targeted Gene		Primer Name	Sequence (5′ to 3′) ^a^	Length (Bases)	Amplicon Size	Tm (°C)	Reference
*Enterobacterales*							
ESBL	*bla*_TEM_ type	TEM-F	GCGGAACCCCTATTTG	16	964 bp	56	12
		TEM-R	ACCAATGCTTAATCAGTGAG	20			
	*bla*_SHV_ type	SHV-F	CTTTACTCGCCTTTATCG	18	827 bp	56	13
		SHV-R	TCCCGCAGATAAATCACCA	19			
	*bla*_CTX-M_ type	CTX-M-F	ATGTGCAGYACCAGTAARGT	20	593 bp	56	14
		CTX-M-R	TGGGTRAARTARGTSACCAGA	21			
	*bla*_OXA-1-like_ type	OXA-1F	ACACAATACATATCAACTTCGC	22	813 bp	56	15
		OXA-1R	AGTGTGTTTAGAATGGTGATC	21			
	*bla*_OXA-2-like_ type	OXA-2F	TTCAAGCCAAAGGCACGATAG	21	702 bp	58	15
		OXA-2R	TCCGAGTTGACTGCCGGGTTG	21			
	*bla*_OXA-10-like_ type	OXA-10F	CGTGCTTTGTAAAAGTAGCAG	21	651 bp	56	15
		OXA-10R	CATGATTTTGGTGGGAATGG	20			
AmpC	*bla_LAT_* type, *bla_CMY_* type, *bla_BIL_* type	CIT-F	TGGCCAGAACTGACAGGCAAA	21	462 bp	64	16
		CIT-R	TTTCTCCTGAACGTGGCTGGC	21			
	*bla*_MOX_ type, *bla*_CMY_ type	MOX-F	GCTGCTCAAGGAGCACAGGAT	21	520 bp	64	16
		MOX-R	CACATTGACATAGGTGTGC	19			
	*bla*_DHA_ type	DHA-F	AACTTTCACAGGTGTGCTGGGT	22	405 bp	64	16
		DHA-R	CCGTACGCATACTGGCTTTGC	21			
	*bla*_ACC_ type	ACC-F	AACAGCCTCAGCAGCCGGTTA	21	346 bp	64	16
		ACC-R	TTCGCCGCAATCATCCCTAGC	21			
	*bla*_MIR_ type, *bla*_ACT_ type	EBC-F	TCGGTAAAGCCGATGTTGCGG	21	302 bp	64	16
		EBC-R	CTTCCACTGCGGCTGCCAGTT	21			
	*bla*_FOX_ type	FOX-F	AACATGGGGTATCAGGGAGAT	21	190 bp	64	16
		FOX-R	CAAAGCGCGTAACCGGATTGG	21			
*Enterococcus faecium*							
Vancomycin resistance	*vanA/B* genes	VanA-F	GGGAAAACGACAATTGC	17	732 bp	62	17
		VanA-R	GTACAATGCGGCCGTTA	17			
		VanB-F	ATGGGAAGCCGATAGTC	17	635 bp	62	17
		VanB-R	GATTTCGTTCCTCGACC	17			
*Staphylococcus aureus*							
Methicillin resistance	*mecA* gene	MecA-F	TCCAGATTACAACTTCACCAGG	22	162 bp	53	18
		MecA-R	CCACTTCATATCTTGTAACG	20			

^a^ For degenerate primers: R = A or G; S = G or C; Y = C or T.
